# Development of potential manufacturing routes for substituted thiophenes – Preparation of halogenated 2-thiophenecarboxylic acid derivatives as building blocks for a new family of 2,6-dihaloaryl 1,2,4-triazole insecticides

**DOI:** 10.1186/1860-5397-3-23

**Published:** 2007-09-04

**Authors:** John W Hull, Duane R Romer, David E Podhorez, Mezzie L Ash, Christine H Brady

**Affiliations:** 1 The Dow Chemical Company, Engineering and Process Sciences, 1710 Building, Midland, MI 48674, USA; 2 The Dow Chemical Company, Chemical Sciences, 1776 Building, Midland, MI 48674, USA; 3 Dow AgroSciences, Indianapolis, IN, USA; 4 The Dow Chemical Company, Process Analytical Sciences, 1897 Building, Midland, MI 48674, USA

## Abstract

**Background:**

Dow AgroSciences has been investigating a new family of functionalized 2,6-dihaloaryl 1,2,4-triazole insecticides featuring specifically targeted insecticidal activities coupled with low mammalian toxicity. With broad spectrum control of both chewing and sap-feeding pests in mind, this family of compounds has been under investigation for aphid, mite, and whitefly control in food crop protection as well as ornamental applications. Two specific targets for development have been the 2,6-dihalo 1,2,4-triazoles XR-693 and XR-906, which require a supply of the halogenated 2-thiophenecarboxylic acid derivatives **1, 2**, and **3** for assembly of the C-ring portion of the triazole products.

**Results:**

Potential manufacturing routes to three halogenated 2-thiophenecarboxylic acid derivatives 4-bromo-3-methyl-2-thiophenecarbonyl chloride **1**, 3,4,5-trichloro-2-thiophenecarbonyl chloride **2**, and 3,4,5-trichloro-2-thiophenecarbonitrile **3** from commercially available thiophene raw materials have been developed and demonstrated on a laboratory scale. A one-pot bromination/debromination procedure developed for 3-methylthiophene gave 2,4-dibromo-3-methylthiophene. Carboxylic acid functionality was then introduced either by a Grignard metallation followed by carbonation with CO_2_, or by a palladium catalyzed carbonylation procedure under CO pressure. The vapor phase chlorination of 2-thiophenecarbonitrile with chlorine gas at 500°C with an average residence time of 6 seconds gave 3,4,5-trichloro-2-thiophenenitrile **3** in a 69% distilled yield, a process that was carried out on a multi-kilogram scale in the laboratory. Finally, a route for the preparation of 3,4,5-trichloro-2-thiophenecarbonyl chloride **2** was developed from tetrachlorothiophene via either a lithiation reaction with *n*-butyllithium in MTBE solvent, or by a previously reported Grignard method using 1,2-dibromoethane as activator, followed by carbonation of the anion with CO_2_ to give the trichloro-2-thiophenecarboxylic acid, which was readily converted to the acid chloride **2** with SOCl_2_.

**Conclusion:**

The successful development of efficient synthetic routes to the halogenated thiophene building blocks 4-bromo-3-methyl-2-thiophenecarbonyl chloride **1**, 3,4,5-trichloro-2-thiophenecarbonyl chloride **2**, and 3,4,5-trichloro-2-thiophenecarbonitrile **3** paved the way for the development of viable commercial processes for XR-693 and XR-906, members of a new class of 2,6-dihaloaryl 1,2,4-triazole insecticides that exhibit selective activity against aphids, mites, and whiteflies coupled with low mammalian toxicity. The process development work for the experimental insecticide target molecules XR-693 and XR-906 will be the topic of a forthcoming paper.

## Background

Since the near world-wide ban on agricultural uses of the chlorinated insecticide DDT which began in the 1970's and 1980's, [1] the search for effective and safe insecticides that exhibit low bio-persistence and low toxicity to birds, mammals, and aquatic life has intensified. The requirement for safe insecticides is driven by a continuing need to control malaria and to feed a growing world population. Indeed, it has been estimated that up to 15% of annual global food crops are lost to insects. [2] Dow AgroSciences has been investigating a new family of functionalized 2,6-dihaloaryl 1,2,4-triazole insecticides featuring specifically targeted insecticidal activity coupled with low mammalian toxicity. [3–6] With broad spectrum control of both chewing and sap-feeding pests in mind, this family of compounds has been under investigation for aphid, mite, and whitefly control in food crop protection as well as ornamental applications. Two specific target molecules for development have been the 2,6-dihalo 1,2,4-triazoles XR-693 and XR-906, each containing a halogenated thiophene moiety on the C-ring portion of the tri-ring system.

**Figure 1:**
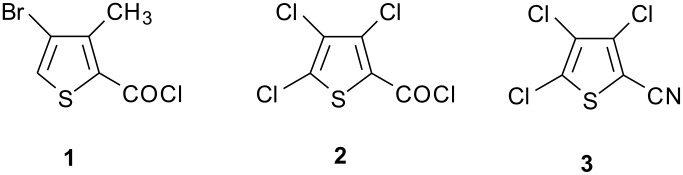
Thiophene structures **1**, **2**, and **3**.

**Figure 2:**
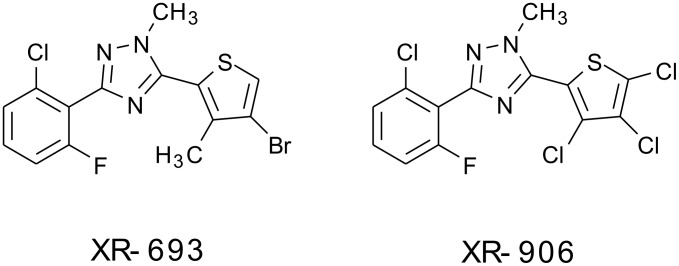
XR-693 and XR-906.

The development of a commercial manufacturing process for these 2,6-dihalo insecticides has required processes for efficient routes to the halogenated 2-thiophenecarboxylic acid derivatives **1–3** (Figure 1), in which the carboxylic acid is functionalized as an acid chloride or nitrile to serve as a handle for attachment to the final triazole ring systems of XR-693 and XR-906 (Figure 2).

In this paper we discuss recent work in the development of potential manufacturing routes to the halogenated thiophene derivatives **1–3** from commercially available and inexpensive thiophene raw materials.

## Bromination Studies. Routes to 4-Bromo-3-methyl-2-thiophenecarbonyl chloride 1

Development of a viable commercial route to the insecticide XR-693 required a supply of 4-bromo-3-methyl-2-thiophenecarbonyl chloride **1** as a building block for attachment of the C-ring. However, neither **1** nor its carboxylic acid precursor were available on a commercial scale, although a 5 Kg research sample of **1** was secured from an intermediates supplier during the course of our study (see Experimental section in Supporting Information File 1). The preparation of the acid precursor of in modest yield from methyl 3-methylthiophene-2-carboxylate via aqueous bromination, followed by hydrolysis of the methyl ester was reported in 1938. [7] Thus, a commercially viable process to **1** was required that could be carried out on a multi-Kg scale or larger.

**Scheme 1:**
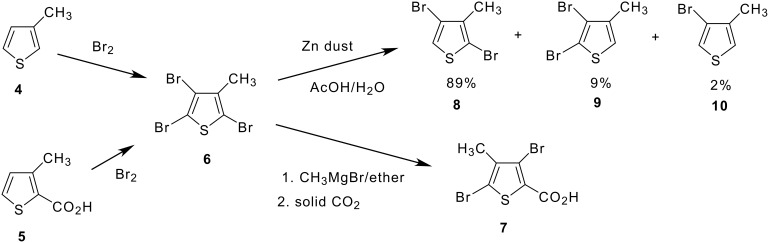
Synthesis and Reactivity of 2,4,5-Tribromo-3-methylthiophene. Initial Bromination Approaches.

**Scheme 2:**
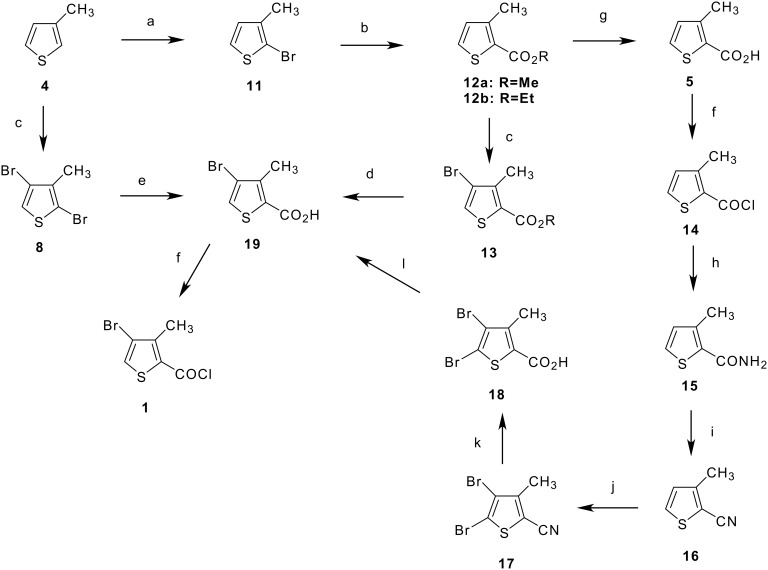
**Routes to 4-Bromo-3-methyl-2-thiopheneacid Chloride, 1, From 3-Methylthiophene, 4**. (a) NBS/AcOH, 64%; (b) Pd(OAc)_2_/DPPP, Na_2_CO_3_, abs EtOH, 33 bar CO; or Mg turnings, THF, then dimethylcarbonate; (c) Br_2_/AcOH, NaOH; 60°C; then Zn dust, 85°C; (d) NaOH,/H_2_O, 91%; (e) MeMgBr (3M in ether), 1,2-DME, 50°C, 5 hr; CO_2_ gas, 5°C; or 10% Pd/C/DPPP cat., Et_3_N/H_2_O, 34 bar CO pressure, 100°C, 59 h; (f) SOCl_2_, cat. DMF, 1,2-DCE, 80°C; (g) NaOH/EtOH; (h) NH_3_/H_2_O; (i) POCl_3_; (j) Br_2_/DMF, 60°C; (k) NaOH/EtOH/H_2_O; (l) Zn dust, AcOH, 100°C; (m) MeOH (to **12a**)

Initial studies of potential viable routes centered upon bromination strategies of the two commercially available 3-methylthiophenes **4** and **5** (Scheme 1). The preparation of **5** has been reported via reaction of 3-methyl-2-thienylmagnesium halides with CO_2_. [8–11] The preparation of the tribromide **6** has also been reported from the bromination of **4**. [11] Our initial approach was to attempt a bromination/debromination sequence on **5** to prepare the acid precursor of **1**, a process that would give **1** from **5** in three chemical steps. However, treatment of **5** with bromine gave tribromide **6** via a bromination/decarboxylation sequence, a procedure that was initially reported in 1953 and used in subsequent synthetic strategies (Scheme 1). [12–15] Tribromide **6** could be reduced somewhat selectively with zinc powder in acetic acid to give a mixture of de-brominated species **8–10**, with the desired 2,4-dibromo isomer **8** formed as 89% of the mixture (Scheme 1). The availability of dibromo intermediate **8** paved the way for insertion of the carboxylic acid functionality at the 2-thienyl position in three chemical steps from commercially available 3-methylthiophene **4** (see discussion below). Interestingly, formation of the Grignard of tribromide **6** by treatment with MeMgBr, [11] followed by treatment with CO_2_ gas led to **7** in an 86% yield, identified by NMR and distinguished from isomer **18** (Scheme 2) by its melting point. This indicates a preferred formation of thienyl Grignard in the undesired position of **6** under these conditions (Scheme 1).

Route selection studies carried out for approaches to **1** are summarized in Scheme 2. The key target was the bromo thiophene-2-carboxylic acid intermediate **19**, which could be readily and cleanly converted to **1** by treatment with thionyl chloride. Scheme 2 shows three potential routes to **19** which were investigated in this study. Initially, the commercially available 3-methylthiophene-2-carboxylic acid **5** was chosen as the starting material. With this approach, the failure of a bromination/debromination scheme to give **19** directly led initially to a more indirect route through the nitrile **16**. The 2-thienylnitrile **16** was prepared from **5** by conversion to the acid chloride **14** followed by treatment with ammonia to give the amide **15**. Dehydration of **15** with POCl_3_ then gave **16** in high-purity. The bromination of **16** was carried out with Br_2_ in DMF, followed by a basic nitrile hydrolysis to give the dibromo acid **18**, which was debrominated with zinc dust in acetic acid to give **19**.

Although this route provided small-scale quantities of **1** for sample studies, a shorter process was desired. Thus, a second approach shown in Scheme 2 was investigated. Mono-bromination of commercially available **4** with NBS gave 2-bromo-3-methylthiophene **11** in a 64% yield. [10] This reaction has also been reported using aqueous bromine. [11] The carboxyl functionality was then introduced by formation of the 2-thienyl Grignard reagent [8–11] followed by treatment with dimethylcarbonate (DMC) to give ester **12a**. Alternatively, a palladium-catalyzed carbonylation under CO pressure in EtOH gave ester **12b**. Unlike **5**, the esters **12** could be subjected to a one-pot bromination/debromination sequence with Br_2_ in acetic acid, followed by Zn dust treatment, to give the 4-bromoester **13**, which was readily hydrolyzed to the acid **19**. This resulted in a five-step chemical process to **1** from commercially available **4**. A four-step route to **1** was also demonstrated from commercially available acid chloride **14** via esterification with methanol to give **12a** [16]. Multi-gram quantities of **1** were prepared on a 5-L laboratory scale using this route (see Supporting Information File 1 for full experimental data).

The shortest, most efficient route to **1** utilized the one-pot bromination/debromination of **4** (Scheme 1 and Scheme 2) to give the 2,4-dibromo intermediate **8**. This proceeded through the unisolated tribromide **6**, which was treated with zinc dust to reduce the 5-bromo position. Although **9** and **10** are co-products (Scheme 1), dibromide **8** is a low-melting solid that can be vacuum distilled to improve purity. Introduction of carboxyl functionality in **8** to give **19** was achieved either by metallation with MeMgBr [11] followed by treatment with CO_2_ gas, or by a palladium catalyzed carbonylation procedure [17–18] under CO pressure in Et_3_N/water using the chelating diphosphine ligand 1,3-bis (diphenylphosphino)propane (DPPP), [19–20] (Scheme 2). The palladium catalyzed carbonylation procedure was also used to prepare esters **12** from **11**. This procedure, [17] which used palladium acetate as the catalyst precursor, was successfully modified in our hands [18] to allow for the use of heterogeneous palladium on carbon catalyst. The ability to use a heterogeneous form of catalyst opens the door for catalyst recycle for an industrially viable process, although carbonylation rates were found to be slower for the heterogeneous catalyst compared to homogeneous palladium acetate. The use of DPPP was required for successful conversion to carbonylation product, even with the use of heterogeneous Pd/C catalyst. Carbonylation of **8** in an Et_3_N/H_2_O solvent system gave acid **19**, while carbonylation of **11** in ethanol gave ester **12b**.

Thus, the most likely commercial scenario for production of **1** is a three chemical step process from **4** via a one-pot bromination/debromination using bromine followed by reduction with zinc dust. This gives bromine substitution at the desired 4 position of the thiophene ring without isolation of the tribromo intermediate **6**. The final low-melting acid chloride **1** was readily purified by vacuum distillation on a 250 g laboratory scale to remove a small amount of tar and gave **1** as a light-yellow colored product.

## Vapor Phase Chlorination Studies. Routes to 3,4,5-Trichloro-2-thiophenecarbonitrile, 3

**Scheme 3:**
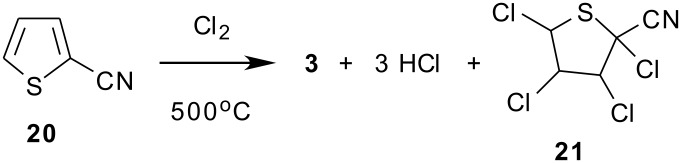
Vapor Phase Chlorination of 2-Thiophenecarbonitrile.

Our investigation of synthetic routes to the 1,2,4-triazole insecticide XR-906 required a supply of the chlorinated thiophenes **2** or **3** as building blocks for attachment of the C-ring portion. Preparation of trichloro-2-thiophenecarbonitrile **3** has been reported starting from 2-chloromethyl-3,4,5-trichlorothiophene. [21] However, since 2-thiophenecarbonitrile **20** was commercially available, we investigated the vapor phase chlorination of this starting material as a potential convenient manufacturing route to **3** that could be carried out on a multi-Kg scale (Scheme 3). [22–23] The vapor phase chlorination of nitrogen heterocycles has been reported. [22] The analogous vapor phase chlorination of thiophene **20** was carried out in the present study using a down flow quartz reactor tube heated with an electric furnace to high temperatures, and filled with an inert support of grade 03 silica gel, which generated a high surface area reaction zone. By feeding a 20% perchloroethylene solution of **20** to the reactor with an excess of chlorine gas at an average reactor residence time of approximately 6 seconds, **3** was obtained as the major product along with minor amounts of the chlorine addition product, thiolane **21**, identified by its mass spectrum. Also present in the crude product were dichlorinated isomers of **20**, a small amount of tetrachlorothiophene, and some hexachloroethane arising from the chlorination of perchloroethylene solvent. A small amount of heavier tars were also formed.

An initial screening program was carried out in which the effect of reaction temperature on the yield of **3** and on the product composition was determined. The results are summarized in Table 1, in which GC analysis of the products obtained at each temperature are compared. A reaction temperature range of 500–630°C was investigated, and the results show that operating the reactor at a lower temperature of 500°C gave a 93% yield of **3** while suppressing the formation of impurities such as **21**. This was a dramatic yield improvement over those obtained at the higher temperatures. The formation of hexachloroethane from perchloroethylene solvent chlorination was more pronounced at lower temperatures, presumably due to longer reactor residence times.

**Table 1:** Vapor Phase Chlorination of 2-Thiophenecarbonitrile **20**: Product Composition as a Function of Reactor Temperature (GC Area % Values)

**Temp (°C)**	630	600	550	500
**Dichloro isomers**	< 5%	6.3%	5.3%	1.7%
**22**	1.1%	1%	1%	<0.5%
**3**	84.9%	83.7%	89.0%	90.5%
**21**	5.7%	3.4%	1.6%	0.6%
**HCE** **^a^**	1.0%	3.5%	4.0%	7.8%
**Yield 3** **^b^**	73%	69%	81%	93%

^a^Hexachloroethane ^b^Based on a GC assay method using 3,4,5,6-tetrachloro- 2-pyridinenitrile as internal standard.

With preliminary studies completed (Table 1), a 4.7 Kg sample of raw material **20** was converted to 8 Kg of crude **3** over a 4-week period using a 1" diameter lab scale chlorination reactor. The average residence time in the reactor was 5–6 seconds. The crude reactor condensate, after neutralization with bicarbonate and solvent evaporation, was vacuum distilled to give 6.4 Kg of **3** having a purity of > 99% by GC area % analysis, representing a 69% distilled yield. The product could also be purified by recrystallization from warm heptane.

The chlorination of **20** was found to be exothermic as noted from internal thermocouples placed inside the reactor tube. An internal exotherm of 20–30°C near the top of the reactor zone was observed. At the end of an extended run, the inert silica gel packing in the reactor tube was blackened, but could easily be poured from the 1" tube.

## Chemistry of Tetrachlorothiophene, 22. Routes to 3,4,5-Trichloro-2-thiophenecarbonyl Chloride, 2

**Scheme 4:**
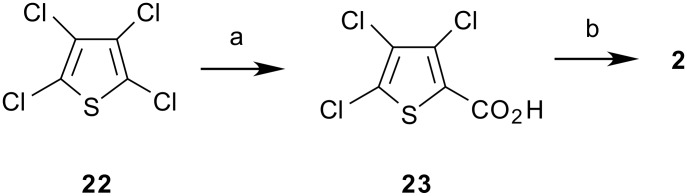
**Synthesis of 3,4,5-Trichloro-2-thiopheneacid Chloride, 2**. (a) 1. n-BuLi/MTBE, -60°C, 2. CO_2_; or 1. Mg/1,2-DBE/THF, 35°C, 2. CO_2_; (b) SOCl_2_, cat. DMF, 1,2-DCE, 80°C

The preparation of tetrachlorothiophene **22** from tetrachloroethylene and H_2_S or from hexachlorobutadiene and sulfur has been reported. [24–26] The synthesis of 3,4,5-trichloro-2-thiophenecarboxylic acid **23** has been reported in an 87% yield from **22** by CO_2_ carbonation of trichloro-2-thienylmagnesium halide, prepared in high yield by the use of a stoichiometric quantity of 1,2-dibromoethane (DBE) as an activator of the magnesium metal (Scheme 4). [19–20] The preparation of 3,4,5-trichloro-2-thiophenecarbonyl chloride **2** from the acid **23** has been reported using an excess of SOCl_2_, followed by the purification of **2** by vacuum distillation. [27]

**Scheme 5:**
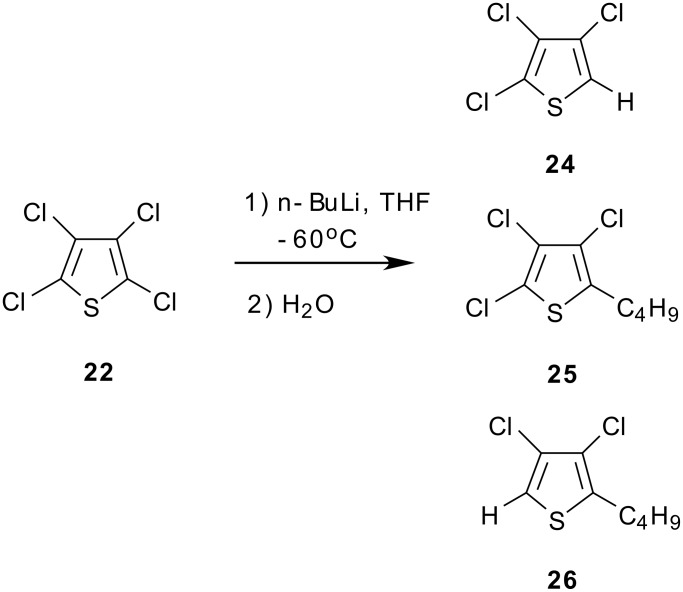
Metal-Halogen Exchange in THF Solvent.

We have found that the preparation of **23** could also be carried out from **22** via metallation with *n*-butyllithium in an ether solvent with sub-zero cooling, followed by quenching of the 2-thienyllithium species with CO_2_. We have found that the choice of solvent for the metal-halogen exchange reaction is important. Diethyl ether was effective, but the preferred choice of solvent for this reaction was found to be methyl *t*-butyl ether, owing to its simplification of reaction work up from the ease of separation of organic and aqueous phases. Interestingly, reaction of **22** with *n*-butyllithium in THF solvent led to a black reaction mixture consisting of undesired byproducts. With n-BuLi, the metallation of **22** in THF at -60°C followed by hydrolysis with water led to a mixture of products that consisted mainly of the trichlorothiophene reduction product **24** arising from protonation of the desired 2-thienyllithium species. However, also present were the butylated thiophenes **25** and **26** (Scheme 5), identified by GC/MS. Thus, in THF the *n*-butyllithium reagent was too reactive, leading to butyl addition products rather than clean metallation to the 2-thienyllithium intermediate.

After successful metallation of **22** and treatment with CO_2_, 3,4,5-trichloro-2-thiophenecarboxylic acid **23** was isolated and dried prior to its conversion to **2** with thionyl chloride. Thus, **2** can be prepared in two chemical steps from the inexpensive chlorinated thiophene **22** using either lithiation or Grignard chemistry. [28]

A comparison of some key manufacturing issues surrounding the two proposed routes to **2** via **23** is summarized in Table 2. The two routes are dramatically different and lead to significantly different yields. At first glance, the lithiation route is clearly superior due to the 92% isolated yield, and the formation of only one equivalent of lithium salt. However, the lithiation route requires sub-zero cooling, and *n*-butyllithium has a significantly higher cost per mole than magnesium metal. The requirement of a -60°C reaction temperature for lithium/chlorine exchange will require a significant capital expenditure in refrigeration equipment, whereas the Grignard method can be carried out by using more conventional manufacturing facilities. Also, the waste disposal of lithium salts can sometimes be problematic. On the other hand, the Grignard route, while using inexpensive magnesium metal, also requires the use of stoichiometric 1,2-dibromoethane (DBE). While DBE is relatively inexpensive, this will result in the generation of nearly three moles of magnesium halide salts per mole of **23** produced. Thus, the low cost of reagents will be at least partly offset by higher waste treatment costs, which are often overlooked in cost analyses of organic processes. [29] For larger volumes of production, the higher yield of the lithiation method will likely prove advantageous, as the increased yields will offset the higher initial investment of refrigeration equipment, as well as the benefits of lower waste volumes generated. For smaller pilot scale operations, the Grignard route may be more advantageous, as it can be carried out in conventional equipment, and in some instances equipment already in place. Additionally, the Grignard route may reap the benefit of additional optimization work that may bring the isolated yield of **23** closer to that obtained with the lithiation route. A careful financial analysis, taking into account all costs, including true waste treatment costs, is required to make a proper selection of the most desirable route.

**Table 2:** Lithiation vs. Grignard Manufacturing Issues for the Preparation of 3,4,5-Trichloro-2-thiophenecarboxylic Acid **23**

**Route**	**Solvent**	**Reagent**	**Temperature (°C)**	**Yield** **23**	**Salt Waste**

Lithiation	MTBE	n-BuLi	-60	92%	1 eq Li salts
Grignard	THF	Mg,/1,2-DBE	36	87%^a^	up to 2.7 eq Mg halide salts

^a^See text reference [28].

## Conclusion

Potential manufacturing routes to halogenated 2-thiophenecarboxylic acid derivatives **1–3** from commercially available raw materials have been developed and demonstrated on a laboratory scale. The halogenated thiophenes with an acid chloride or nitrile functional group at the 2-position are key building blocks for a new family of 1,2,4-triazole insecticides. A one-pot bromination/debromination procedure developed for 3-methylthiophene gave 2,4-dibromo-3-methylthiophene, which was a key intermediate for conversion to 4-bromo-3-methyl-2-thiophenecarbonyl chloride **1**. Carboxylic acid functionality was introduced either by a Grignard reaction followed by carbonation with CO_2_, or a palladium catalyzed carbonylation procedure under CO pressure.

The vapor phase chlorination of 2-thiophenenitrile at 500°C gave 3,4,5-trichloro-2-thiophenecarbonitrile **3** in a 69% distilled yield on a multi-kilogram scale, carried out using a 1" diameter lab-scale reactor tube over a four week campaign. Finally, a route for the preparation of 3,4,5-trichloro-2-thiophenecarbonyl chloride **2** was developed from tetrachlorothiophene via either a lithiation reaction with *n*-butyllithium in MTBE solvent, or by a previously reported Grignard method using 1,2-dibromoethane as activator, followed by carbonation of the anion with CO_2_ to give the 2-thiophenecarboxylic acid, which was readily converted to **2** with SOCl_2_.

The successful development of efficient synthetic routes to the halogenated thiophene building blocks **1–3** has paved the way for a development program for a new class of 1,2,4-triazole insecticides XR-693 and XR-906. A summation of the process development work of these two new insecticides will be the focus of a forthcoming publication.

## Experimental

See Supporting Information File 1 for full experimental data

## Supporting Information

File 1 Development of potential manufacturing routes for substituted thiophenes – Preparation of halogenated 2-thiophenecarboxylic acid derivatives as building blocks for a new family of 2,6-dihaloaryl 1,2,4-triazole insecticides. A detailed experimental section for the synthesis of thiophenes **1, 2,** and **3** and their key intermediates is provided.
