# Supplementary material for: Development of potential manufacturing routes for substituted thiophenes – Preparation of halogenated 2-thiophenecarboxylic acid derivatives as building blocks for a new family of 2,6-dihaloaryl 1,2,4-triazole insecticides
Source: Beilstein J Org Chem. 2007 Sep 4;3:23. doi: 10.1186/1860-5397-3-23 (PMC2148045; doi:10.1186/1860-5397-3-23)
Supplement: File 1 — Development of potential manufacturing routes for substituted thiophenes – Preparation of halogenated 2-thiophenecarboxylic acid derivatives as building blocks for a new family of 2,6-dihaloaryl 1,2,4-triazole insecticides. A detailed experimental section for the synthesis of thiophenes 1, 2, and 3 and their key intermediates is provided. [file Beilstein_J_Org_Chem-03-23-s001.doc]

**Additional Material**

# Development of potential manufacturing routes for substituted thiophenes - Preparation of halogenated 2-thiophenecarboxylic acid derivatives as building blocks for a new family of 2,6-dihaloaryl 1,2,4-triazole insecticides

John W. Hull, Jr.*^1^, Duane R. Romer^2^, David E. Podhorez^1^, Mezzie L. Ash^3^ and Christine H. Brady^4^

Address: ^1^The Dow Chemical Company, Engineering and Process Sciences, 1710 Building, Midland, MI 48674, United States, ^2^The Dow Chemical Company, Chemical Sciences, 1776 Building, Midland, MI 48674, United States, ^3^Dow AgroSciences, Indianapolis, IN, United States, retired and ^4^The Dow Chemical Company, Process Analytical Sciences, 1897 Building, Midland, MI 48674, United States

Email: John W. Hull, Jr.* - [jwhull@dow.com](mailto:jwhull@dow.com); Duane R. Romer - [drromer2@dow.com](mailto:drromer2@dow.com); David E. Podhorez – [depodhorez@dow.com](mailto:depodhorez@dow.com); Mezzie L. Ash – [mezzie1948@sbcglobal.net](mailto:mezzie1948@sbcglobal.net); Christine H. Brady – [chbrady@dow.com](mailto:chbrady@dow.com)

*Corresponding author

# Experimental Section

**General.** *Note: Carbon monoxide should only be used in a well ventilated area and with adequate detection systems designed to alarm well below the lethal limit of 300 ppm CO.* Reactions with CO were carried out in a well ventilated explosion proof cubicle with a CO detection and alarm system. Reaction vessels were standard pressure reactors of 316 SS or Hastelloy construction with overhead agitation. 3-Methylthiophene **4** was purchased from Sigma Aldrich or Lancaster Chemical. [1] 2-Bromo-3-methylthiophene **11** was prepared as noted below and was also purchased from Lancaster Chemical. [1] 3-Methyl-2-thiophenecarbonyl chloride **14** and 4-bromo-3-methyl-2-thiophenecarbonyl chloride **1** were purchased in 5 Kg research quantities from Shasun Chemical and Drugs, Ltd. of India. [2] Five-kilogram samples of tetrachlorothiophene **22** and 2-thiophenecarbonitrile **20** were purchased from Lancaster Chemical. [1] Silica gel (grade 03, 8 mesh) used in the vapor phase chlorination reactions was obtained from Sigma Aldrich. Solvents, palladium catalysts, and the phosphine ligand 1,3-bis(diphenylphosphino)propane (DPPP) were obtained from standard commercial sources. Intermediate 2,4,5-tribromo-3-methylthiophene **6** was prepared by the bromination of either 3-methylthiophene **4** or 3-methyl-2-thiophenecarboxylic acid **5** according to published procedures. [3,4,5,6,7] All reactions except those noted were carried out in standard laboratory glassware and heated using an electric heating mantle with electronic controller.

**2-Bromo-3-methylthiophene, 11.** [3,8] To a solution of glacial AcOH (100 mL), acetic anhydride (4.0 g, 0.039 mol), and 3-methylthiophene **4** (9.82 g, 0.100 mol) as a stirred mixture was added NBS (19.6 g, 0.11 mol) in one portion with a slight exotherm to 44 ^o^C. After 20 min the reaction mixture was poured onto ice, neutralized with sodium bisulfite and extracted with ether. The ether layer was washed with water and concentrated to give 11.3 g (64%) of 2-bromo-3-methylthiophene **11** as a tan colored liquid in 88% purity by GC area % analysis: ^1^H NMR (CDCl_3_) δ 7.1 (d, 1H, J=5.7 Hz), 6.7 (d, 1H, J=5.9 Hz), 2.2 (s, 3H).

**One Pot Synthesis of 2,4-Dibromo-3-methylthiophene, 8.** A mixture of 3-methylthiphene **4** (50.0 g, 0.509 mol), glacial AcOH (400 mL) and NaOAc (132 g, 1.60 mol) was stirred and heated to 60 ^o^C. Bromine (260 g, 1.63 mol) was then added dropwise at such a rate that kept the temperature below 85 ^o^C. The mixture was heated at 85 ^o^C for 5 h to complete the conversion to 2,4,5-tribromo-3-methylthiophene **6**, then water (85 g) was added and the reaction temperature was dropped to 60 ^o^C. Zinc dust (48.1 g, 0.73 mol) was carefully added in 5-10 g portions so as to keep the temperature below 85 ^o^C. The mixture was heated to 100 ^o^C for 8 h, then cooled to 25 ^o^C. Water (350 mL) and hexanes (500 mL) were then added. The mixture was stirred for 30 min, the organic layer was separated, washed with water (150 mL), then saturated NaHCO_3_ solution (150 mL), then concentrated to give an orange oil. This oil was distilled (b.p. 80 ^o^C @ 0.3 mm Hg) through a 6” jacketed Vigreux column to give 107 g (82% wt.) of a yellow oil which slowly solidified. GC area % analysis showed the product to be a mixture of 2,4-dibromo-3-methylthiophene **8** (89%), 4,5-dibromo-3-methylthiophene **9** (9%), and 4-bromo-3-methylthiophene **10** (2%).

**3-Methyl-2-thiophenecarboxylate Esters, 12**. *A. Grignard Method*. 2-Bromo-3-methylthiophene **11** (10.0 g, 0.0565 mol) was added dropwise over a 60 min period to magnesium turnings (1.72 g, 0.0706 mol) in anhydrous THF. The reaction temperature was kept below 40 ^o^C with a cooling bath, and after the addition was complete the mixture was stirred at 25 ^o^C for another 60 min. Dimethylcarbonate (7.63 g, 0.0847 mol) was added dropwise over 5 min and the mixture was stirred for 4 h. After quenching with 6N HCl and extraction with EtOAc, the organic phase was washed with water, extracted with aqueous NaHCO_3_, and evaporated to give 7.38 g (84% wt.) of methyl 3-methyl-2-thiophenecarboxylate **12a** as a light yellow oil.

*B. Pd Catalyzed Carbonylation Method.* [9] A mixture of 2-bromo-3-methylthiophene **11** (26.4 g, 0.149 mol), Pd(OAc)_2_ (0.213 g, 0.00095 mol), 1,3-bis(diphenylphosphino)propane (DPPP, 0.49 g, 0.0012 mol), Na_2_CO_3_ (21.1 g, 0.20 mol), and 200 mL absolute EtOH were weighed into a stainless steel pressure reactor. After purging with N_2_, the vessel was pressurized to 34 bar with CO and stirred at 120 ^o^C for 36 h. After cooling and venting, the reactor contents were filtered and evaporated to an oil/solid mixture. This residue was extracted with 1,2-dichloroethane/water, and the organic phase was evaporated to give an oil which was filtered to give 20.7 g (81% wt.) of ethyl 3-methyl-2-thiophenecarboxylate **12b** as an orange oil: ^13^C NMR {^1^H} CDCl_3_: δ 162.8, 145.9, 131.7, 129.9, 127.0, 60.6, 15.9, 14.4 ppm.

*C. Methanolysis of* **14.** [10] 3-Methyl-2-thiophenecarbonyl chloride **14** (Shasun, 1238 g, 7.7 mol) was added dropwise over a 4 h period to 2500 mL of stirring MeOH. The reactor was vented to a 15% NaOH solution to scavenge the HCl gas evolved. During the addition the solution temperature rose to 45 ^o^C. The methanol was evaporated and the product was kept under a vacuum in a 60 ^o^C water bath to remove residual solvent and HCl. The resulting product methyl 3-methyl-2-thiophenecarboxylate **12a** (1181 g, 98% wt.) was obtained as a light yellow oil: ^13^C NMR {^1^H} CDCl_3_: δ 163.2, 146.2, 131.7, 130.0, 126.5, 51.6, 15.9 ppm; ^1^H NMR (CDCl_3_) δ 7.3 (d, 1H), 6.8 (d, 1H), 3.8 (s, 3H), 2.5 ppm (s, 3H).

**Methyl 4-bromo-3-methyl-2-thiophenecarboxylate, 13**. Aqueous 50% NaOH (520 mL, 9.9 mol) was added carefully in portions over an 11 min period to 2200 mL of glacial AcOH, resulting in an exotherm to 99 ^o^C. Methyl 3-methyl-2-thiophenecarboxylate **12a** (415 g, 2.7 mol) was added, and Br_2_ (480 mL, 9.3 mol) was added dropwise over 1.25 h at a rate that maintained a reactor temperature below 100 ^o^C (the solution darkens in color above 100 ^o^C). After Br_2_ addition was complete, the reactor was maintained at 88 ^o^C (the dibromo intermediate was found to crystallize from solution below 85 ^o^C). The heat was removed, and Zn powder (600 g, 9.2 mol) was added in 30-g portions over a 3.25 h period at such a rate so as to maintain the reaction temperature below 100 ^o^C. After the Zn powder addition was complete, heating was re-applied to the reactor to maintain an 80 ^o^C temperature. The solution was hot filtered through Celite to remove residual Zn particles, and the filtrate was poured with stirring into 12.5 L of cold water. The precipitate was collected on a fritted glass funnel and rinsed with water. The wet cake was dissolved in 1.5 L of 1,2-dichloroethane and filtered again through Celite to remove small amounts of remaining Zn particles. The filtrate was evaporated to give 541 g (85% wt.) of methyl 4-bromo-3-methyl-2-thiophenecarboxylate **13** as an orange oil that crystallized on cooling, m.p. 49-57 ^o^C, and having a GC area % purity of 97%: ^13^C NMR {^1^H} CDCl_3_: δ 162.1, 144.6, 127.6, 127.1, 115.3, 52.0, 15.4 ppm (also contains a 1,2-dichloroethane signal at 43.5 ppm); ^1^H NMR (CDCl_3_) δ 7.3 (s, 1H), 3.8 (s, 3H), 2.5 ppm (s, 3H), and a small singlet at 3.7 ppm from residual 1,2-dichloroethane.

**4-Bromo-3-methyl-2-thiophenecarboxylic Acid, 19**. *A. From Ester Hydrolysis.* To a solution of 50% NaOH (97 mL, 1.9 mol) in 1600 mL of water was added methyl 4-bromo-3-methyl-2-thiophenecarboxylate **13** (277 g, 1.18 mol) and the slurry was heated at 65 ^o^C for 1 h. The resulting orange solution was filtered through Celite, diluted with an additional 2 L of water, and 37% aqueous HCl (178 mL, 2.15 mol) was added dropwise to precipitate the acid, resulting in a thick white slurry. The solid was collected on a funnel and rinsed with water, and dried overnight in a vacuum oven at 65^o^C to give 238 g (91% wt.) of 4-bromo-3-methyl-2-thiophenecarboxylic acid **19** as a white solid, m.p. 189-190 ^o^C (Lit. [11] 188-189 ^o^C): ^13^C NMR {^1^H} (acetone-d_6_): δ 164.0, 144.5, 129.0, 128.8, 115.3, 15.2 ppm;); ^1^H NMR (acetone-d_6_) δ 7.8 (s 1H), 2.5 ppm (s, 3H).

*B. From Grignard Reaction*. 2,4-Dibromo-3-methylthiphene **8** (27.1 g, 0.106 mol) was dissolved in 135 mL of anhydrous 1,2-dimethoxyethane, and MeMgBr (44.1 mL of a 3.0 M solution, 0.132 mol) was added over a 30-min period, followed by heating at 50 ^o^C for 5 h. The mixture was cooled to 5 ^o^C and CO_2_ gas (20 g, 0.45 mol) was slowly bubbled into the solution at a rate such that the temperature remained below 10 ^o^C. The mixture was warmed to 25 ^o^C and stirred overnight. After concentrating to dryness, the residue was slurried with water and 6 N HCl, and the resulting white solid was collected by filtration. The product was washed with 6 N HCl, then with water, and then vacuum dried to give 19.6 g (84% wt.) of 4-bromo-3-methyl-2-thiophenecarboxylic acid **19**. The product could be recrystallized from a solvent mixture of methyl-*t*-butyl ether/hexanes to increase the isomeric purity.

*C. Pd Catalyzed Carbonylation Method.* [9] 2,4-Dibromo-3-methylthiphene **8** (5.40 g of 73% purity, 0.015 mol), 10% Pd/C powder (0.1193 g, 0.112 mmol), 1,3-bis(diphenylphosphino)propane (DPPP, 0.13 g, 0.32 mmol), triethylamine (25 mL, 0.18 mol) and 25 mL of water were added to a pressure reactor. After purging with N_2_, the reactor was pressurized with CO to 34 bar and heated at 100 ^o^C with stirring for 59 h. During this time a slow but steady consumption of CO was observed from the drop in reactor pressure. The reactor was cooled, vented, and the contents diluted with water. This mixture was filtered through Celite to remove catalyst, and 50% NaOH (5.0 g, 0.063 mol) was added to the light orange filtrate. The solution was concentrated to remove triethylamine, and filtered again through Celite. The yellow filtrate was acidified with 37% HCl and the white precipitate was collected by filtration and washed with water. The solid was recrystallized from 60/40 ethanol/water to give 2.05 g (58% wt.) of 4-bromo-3-methyl-2-thiophenecarboxylic acid **19**, mp 176-180 ^o^C, having an HPLC area % purity of 96%. The observed melting point was lower than the reported literature melting point of 188-189^o^C [11] most likely due to the presence of water and solvent.

*D. From Zn Reduction of* **18***.* A mixture of 4,5-dibromo-3-methyl-2-thiophenecarboxylic acid **18** (0.85 g, 2.8 mmol), acetic acid (12 mL), water (2.5 mL) and Zn dust (0.22 g, 3.5 mmol) was heated at 100 ^o^C for 2 h. The mixture was cooled to 25 ^o^C and poured into ice water (50 mL). The solids were collected by filtration and dried in air overnight to give 0.55 g (89% wt.) of 4-bromo-3-methyl-2-thiophenecarboxylic acid **19**: m.p. 188-189 ^o^C; ^1^H NMR (CDCl_3_) δ 7.5 (s, 1H), 2.57 ppm (s, 3H); 98% purity by HPLC area %.

**4-Bromo-3-methyl-2-thiopheneacid Chloride, 1**. 4-Bromo-3-methyl-2-thiophenecarboxylic acid **19** (237 g, 1.07 mol), 2000 mL of 1,2-dichloroethane, DMF (4.1 mL, 0.05 mol), and SOCl_2_ (94 mL, 1.3 mol) were stirred together and brought to reflux temperature. The reactor was vented to a SO_2_/HCl scavenging solution prepared by mixing 210 mL 50% NaOH (4.02 mol) into 1.5 L water. The slurry became homogeneous at 50 ^o^C, and the solution reflux temperature gradually increased as SO_2_ and HCl were purged from the solution. The orange solution was heated at reflux temperature (83 ^o^C) for 1 h, then cooled to 25 ^o^C and concentrated to give 285 g of a dark orange oil. This product was purified by vacuum distillation through a short-path distillation head (b.p. 98 ^o^C @ 0.1 mm Hg), while applying hot water to the condenser to prevent the distillate from freezing, to give 250 g of 4-bromo-3-methyl-2-thiophenecarbonyl chloride **1** as a light yellow oil (97% wt.) which crystallized upon standing, m.p. 55-59 ^o^C: ^13^C NMR {^1^H} CDCl_3_: δ 158.4, 148.4, 133.2, 132.1, 117.2, 16.4 ppm; ^1^H NMR (CDCl_3_) δ 7.6 (s, 1H), 2.5 (s, 3H).

**3-Methyl-2-Thiophenecarboxamide, 15**. 1,2-Dichloroethane (280 mL), DMF (0.05 mL), 3-methyl-2-thiophenecarboxylic acid **5** (21.3 g, 0.149 mol), and SOCl_2_ (71.4 g, 43.8 mL, 0.6 mol) were heated at reflux temperature for 2 h with stirring. The solvent was then removed under vacuum to give 24 g of 3-methyl-2-thiophenecarbonyl chloride **14**. This product was dissolved in 100 mL of CH_2_Cl_2_ and added to a solution of concentrated NH_4_OH (110 mL) in 300 mL CH_2_Cl_2_ with ice/salt bath cooling, at such a rate so as to maintain the reaction temperature below –5 ^o^C. The mixture was stirred for 20 min, separated, and the aqueous phase was extracted with another 200 mL of CH_2_Cl_2_. The combined organic layers were washed with water, then brine, and the solvent removed to give 21.2 g of 3-methyl-2-thiophenecarboxamide **15** as an off white solid, m.p. 123-124^o^C; 99.6% pure by GC area %; ^1^H NMR (CDCl_3_) δ 7.3 (d, 1H), 6.9 (1H), 5.8 (s, b), 2.5 ppm (s, 3H).

**3-Methyl-2-thiophenecarbonitrile, 16.** 3-Methyl-2-thiophenecarboxamide **15** (18.6 g, 0.13 mol) was mixed with an excess of POCl_3_, and the mixture heated at reflux temperature for 2 h. The excess POCl_3_ was removed under vacuum and the residue dissolved in 500 mL of 1,2-dichloroethane, washed (carefully!) with water, then brine, and the solvent removed to give 23 g of crude 3-methyl-2-thiophenecarbonitrile **16**. This material was passed through a plug of silica gel with CH_2_Cl_2_ and the solvent evaporated to give 16.2 g (100%) of **16** as a light tan oil: ^1^H NMR (CDCl_3_) δ 7.4 (d, J=4.8 Hz, 1H), 6.9 (d, J=5.4 Hz, 1H), 2.4 ppm (s, 3H); 97% pure by HPLC area %.

**4,5-Dibromo-3-methyl-2-thiophenecarbonitrile, 17**. To a solution of 3-methyl-2-thiophenecarbonitrile **16** (16.0 g, 0.13 mol) in DMF (35 mL) was added, dropwise, two equivalents of Br_2_ (41.5 g, 0.26 mol) over a 45 min period, which raised the temperature to 57 ^o^C. After the addition was complete, the mixture was heated at 60 ^o^C for 20 h, and quenched into ice containing sodium bisulfite. The solid was collected by filtration, dissolved in CH_2_Cl_2_, washed with water, and the solvent evaporated to give 28.3 g (78% wt.) of 4,5-dibromo-3-methyl-2-thiophenecarbonitrile **17** as a tan colored solid: m.p. 80-81 ^o^C; ^1^H NMR (CDCl_3_) δ 2.45 (s); 97% purity by GC area %.

**4,5-Dibromo-3-methyl-2-thiophenecarboxylic acid, 18**. 4,5-dibromo-3-methyl-2-thiophenecarbonitrile **17** (26.2 g, 92.2 mmol), EtOH (175 mL), water (175 mL), and NaOH (18.6 g, 466 mmol) were stirred together at 80 ^o^C for 1 h. The solvent was removed, the solids were collected by filtration, dissolved in water, and washed with CH_2_Cl_2_. The water layer was acidified with 37% HCl, the white solid was collected by filtration and dried under vacuum at 60 ^o^C to give 25.9 g (94% wt.) of 4,5-dibromo-3-methyl-2-thiophenecarboxylic acid **18**: m.p. 225-226 ^o^C; ^1^H NMR (CDCl_3_) δ 2.5 (s).

**3,5-Dibromo-4-methyl-2-thiophenecarboxylic Acid, 7.** Ether (30 mL) and 2,4,5-tribromo-3-methylthiophene **6** (14.7 g, 43.9 mmol) were added to a 250 mL glass roundbottom flask. To the stirred solution was added dropwise MeMgBr (50 mL of a 3 M solution in ether, 150 mmol). The resulting solution was stirred at 25 ^o^C for 7 h. The solution was poured onto a slurry of dry ice in 200 mL ether and was allowed to warm to ambient temperature overnight under a slow stream of N_2_. The resulting solids were slurried in ether, collected by filtration, then slurried in water and the pH lowered to 2 with aqueous HCl. The solids were collected by filtration and vacuum dried at 60^o^C overnight to give 11.3 g (86% wt.) of 3,5-dibromo-4-methyl-2-thiophenecarboxylic acid **7**: m.p. 212-214 ^o^C (Lit. [11] 216-217 ^o^C); ^1^H NMR (CDCl_3_) δ 2.3 (s).

**Vapor Phase Preparation of 3,4,5-Trichloro-2-thiophenecarbonitrile, 3.** The down flow vapor phase reactor consisted of a 1” diameter by 23” length quartz tube heated with a 3-zone electric furnace controlled by three independent electronic controllers. Temperature control thermocouples were attached to the outside of the quartz tube with high-temperature tape; thus the furnace temperature control was maintained at the reactor tube skin. Temperature monitoring thermocouples were placed in a small inside tube at the center of the reactor tube to monitor internal reactor zone temperatures. A 6” preheater tube was mounted on top of the reactor tube and controlled at a skin temperature of 200 ^o^C with an electric heat tape and controller. The preheater tube was filled with quartz chips. The packing material inside the reactor tube was silica gel grade 03, mesh size number 8, obtained from Aldrich. An FMI pump was used to deliver a controlled rate of liquid feed solution to the preheater tube. Chlorine gas from a cylinder was metered into the reactor through a calibrated rotometer. The perchloroethylene product solution was condensed at the bottom of the reactor tube into a receiving vessel using a static air cooling condenser. After condensing the liquid product, the reactor gaseous effluent containing Cl_2_ and HCl was passed through a solution of 15% NaOH.

*Great care should be exercised during the handling of Cl_2_ gas. All operations were carried out in an efficient fume hood. Safety features were built into the reactor system to eliminate the danger of release of Cl_2_ and HCl gases. The Cl_2_ feed line was fitted with an electrically actuated valve powered through a cutoff box, which in turn was interfaced with a Cl_2_ detector. This allowed for the automatic shutoff of organic and Cl_2_ feeds in the event of a loss of hood ventilation or the detection of Cl_2_ gas inside the hood.*

A 20% solution of 2-thiophenecarbonitrile **20** (3633 g, 33.3 mol) in perchloroethylene solvent was pumped into the vapor phase reactor held at 500 ^o^C at a rate of 0.8 g/min (1.47 mmol/min), with a Cl_2_ flow rate of 0.61 g/min (8.60 mmol/min). A N_2_ co-feed of 73 mL/min was also used. A total of 18.2 Kg of perchloroethylene solution was fed to the reactor over a 3-week period. The product solution was washed with aqueous Na_2_CO_3_, followed by solvent removal to give 9103 g of crude 3,4,5-trichloro-2-thiophenecarbonitrile **3** as an orange oil which readily crystallized on cooling. Product assay using a GC internal standard method indicated a purity of 69% **3** (89% yield).

Crude **3** was vacuum distilled at 0.5 mm Hg through a 1” diameter 30-plate Oldershaw column using a simple distillation head and a hot water condenser to maintain a liquid state in the distillate. Fractions were taken at intervals, saving those having a GC area % purity of 99% or greater. Lights cuts containing some dichloro isomers were redistilled to obtain additional high purity **3**. From a total of 4714 g of **20** fed to the reactor, a total of 6360 g of **3** having a GC area % purity of at least 99% was obtained, representing a distilled yield of 69%. For samples of analytical purity, the product could be recrystallized from warm heptane to give white needles: m.p. 63-65 ^o^C (Lit. [12] 60 ^o^C), ^13^C NMR {^1^H} (CDCl_3_) δ 134.6, 131.8, 125.4, 110.5, 103.5 ppm.

**3,4,5-Trichloro-2-thiophenecarboxylic Acid, 23.** *A. From Lithiation of Tetrachlorothiophene.* A solution of 22.2 g (0.1 mol) of tetrachlorothiophene **22** in 150 mL of methyl *t*-butyl ether was cooled to below –60 ^o^C with an acetone/dry ice bath. To this slurry was added 45 mL of a 2.5 M solution in hexanes (0.113 mol) of *n*-butyllithium over 20 min. The mixture was allowed to stir near –50 ^o^C for 30 min. Dry CO_2_ was bubbled into the solution while allowing the reaction mixture to warm to 25 ^o^C over a 30-min period. The reaction mixture was diluted with 150 mL of water. A precipitate initially formed which quickly redissolved. The aqueous layer was separated and acidified with 1N HCl. The slurry was stirred for 1 h and the solid was filtered, washed twice with water and twice with hexanes. The solid was vacuum dried at 40 ^o^C to give 21.3 g (92% wt.) of 3,4,5-trichloro-2-thiophenecarboxylic acid **23** as a light tan powder, m.p. 226-227 ^o^C (Lit. [13] 225-226 ^o^C).

*B. From Grignard Reaction of* **22***.* The formation of 3,4,5-trichloro-2-thiophenecarboxylic acid **23** was prepared from tetrachlorothiophene **22** via Grignard chemistry and 1,2-dibromoethane as activator according to reported procedures. [14]

**3,4,5-Trichloro-2-thiophenecarbonyl Chloride, 2.** [15] A mixture of 3,4,5-trichloro-2-thiophenecarboxylic acid **23** (20.4 g, 0.088 mol), SOCl_2_ (7.3 mL, 0.1 mol), and DMF (0.2 mL) in 80 mL of 1,2-dichloroethane was heated at reflux temperature for 3 h. The reaction mixture became homogeneous after 1 h of heating. The solution was cooled to 25 ^o^C, concentrated and placed under high vacuum to remove residual solvent, giving 22.0 g (98% wt.) of 3,4,5-trichloro-2-thiophenecarbonyl chloride **2** as an oil which solidified on standing to an off-white powder; m.p. 37-41 ^o^C, having a GC area % purity > 99%.

# References for Experimental Section

1. [www.alfa.com](http://www.alfa.com/)
2. [www.shasun.com](http://www.shasun.com/)
3. Steinkopt, Jacob: *Justus Liebigs Ann. Chem. 1935*, **515**:273.
4. Melles JL, Backer HJ: *Recl. Trav. Chim. Pays-Bas* *1953*, **72**:314.
5. Zwanenburg DJ, Wynberg H: *Recl. Trav. Chim. Pays-Bas* *1967*, **86**:589.
6. Morel J, Pastour P: *Bull. Soc. Chim. Fr. 1968*, 737.
7. Zwanenburg DJ, Wynberg H: *Recl. Trav. Chim. Pays-Bas* *1969*, **88**:321.
8. Blanchette, Brown: *J. Amer. Chem. Soc. 1951*, **73**:2779.
9. Hull JW Jr, Wang C: *Heterocycles* *2004*, **63**:411.
10. Aitken RA, Bibby MC, Bielefeldt F, Double JA, Laws AL, Mathieu AL, Ritchie RB, Wilson DWJ: *Archiv. der Pharm.* *1998*, **331**:405.
11. Steinkopt, Nitschke: *Justus Liebigs Ann. Chem. 1938*, **536**:135.
12. Akopyan AN, Kon’kova SG, Safaryan, AA: *J. Org. Chem. USSR (Engl. Trans.) 1979*, **15**:933.
13. Rahman MT, Gilman H: *J. Indian Chem. Soc. 1976*, **53**:269-271.
14. Rahman MT, Smith MR Jr,Webb AF, Gilman H: *Organometallics in Chemical Synthesis 1970/71*, **1**:105.
15. Trinh VQ, Pillon D: German Pat DE 1813195 (*Chem. Abstr.* **72**:12555j).
